# Correlation between inflammatory cytokine expression in paraspinal tissues and severity of disc degeneration in individuals with lumbar disc herniation

**DOI:** 10.1186/s12891-023-06295-z

**Published:** 2023-03-14

**Authors:** Xiaolong Chen, Yongjin Li, Wei Wang, Peng Cui, Yu Wang, Shibao Lu

**Affiliations:** Department of Orthopaedics, Xuanwu Hospital Capital Medical University, Changchun Street 45, Xicheng District, 100032 Beijing, China

**Keywords:** Lumbar disc herniation, Percutaneous endoscopic lumbar discectomy, Multifidus muscle, Fat infiltration, Cross-sectional area, Inflammatory cytokine

## Abstract

**Purpose:**

Previous animal studies have discovered dysregulation of the local inflammatory state as a novel mechanism to explain structural changes in paraspinal muscles in association with disc degeneration. This study aimed to determine whether the expression of inflammatory genes in the multifidus muscle (MM) differs between individuals with disc degeneration and non-degeneration, which may cause changes in the cross-sectional area (CSA) of paraspinal muscles and clinical outcomes.

**Methods:**

Muscles were procured from 60 individuals undergoing percutaneous endoscopic lumbar discectomy for lumbar disc herniation (LDH). Total and functional CSAs and fatty degeneration of paraspinal muscles on ipsilateral and unilateral sides were measured. Gene expression was quantified using qPCR assays. Paired t-test and Pearson’s correlation analysis were used to compare the mean difference and associations, respectively.

**Results:**

There were significant differences in total CSAs of paraspinal muscles and functional CSA and fatty degeneration of MM between ipsilateral and unilateral sides. Participants in the disc degeneration group displayed higher fat infiltration in MM. The expression of TNF was moderately correlated with total CSAs of paraspinal muscles and functional CSA and fatty degeneration of MM. The expression of IL-1β was strongly correlated with the total and functional CSA of MM. The expression of TGF-β1 was moderately correlated with the functional CSA of MM. The expression of TNF, IL-1β, and TGF-β1 was moderate to strongly correlated with clinical outcomes.

**Conclusion:**

The results show that there were differences in the characteristics of paraspinal muscles between the ipsilateral and unilateral sides, which were affected by disc degeneration and the degree of fat infiltration. High-fat filtration and reduction of CSA of MM are associated with inflammatory dysfunction. There was evidence of a dysregulated inflammatory profile in MM in individuals with poor clinical outcomes.

## Introduction

Paraspinal muscles, as the action muscles of the back, are responsible for stabilizing and moving the lumbar vertebral column, which includes the iliopsoas, multifidus muscle (MM), quadratus lumborum, and erector spinae muscle. Several recent studies have investigated the qualitative (e.g., fatty infiltration [1]) and quantitative (e.g., fiber-type transformation [2] and cross-sectional area (CSA) [3]) changes in paraspinal muscles in association with the degeneration of intervertebral discs (IVDs) (e.g., lumbar disc herniation (LDH)). These structural changes in paraspinal muscles also play an important role in low back pain (LBP). Three main mechanisms have been revealed for causing the structural changes in paraspinal muscles, including muscle disuse atrophy, muscle denervation, and dysregulated inflammatory response. Animal studies have indicated that the dysregulation of local inflammatory activity is the main potential contributor to fat and connective tissue accumulation in paraspinal muscles in association with IVD lesions/injuries (e.g., spontaneous degeneration of IVD [4, 5] and experimental IVD injury [2]), which includes the inhibition of paraspinal muscle activation during the acute phase [6] and the loss of slow-twitch muscle fibres, connective tissue accumulation, and fatty infiltration during the subacute and chronic phases of paraspinal muscle atrophy. However, there is a lack of investigation of the relationship between different grades of IVD degeneration in Pfirrmann scores and the structural changes of paraspinal muscles (e.g., increased fatty infiltration and reduced CSA) [7]. Therefore, investigation of the relationship in patients with LDH is required.

Interestingly, an active process mediated by an inflammatory response has been revealed in different pathologies in paraspinal muscles. Animal models provide evidence for proinflammatory cytokine expression (e.g., tumor necrosis factor (TNF)) [8, 9] and localization and polarization of macrophages in MM adipose tissue that could not only drive the accumulation of fibrosis [10] but also associate muscle fiber changes [2, 11]. A recent human study revealed that a dysregulated inflammatory response is associated with increased fatty infiltration in MM in patients with LDH [12]. However, the association between the changes in the CSA of paraspinal muscles and inflammatory dysregulation remains unclear. It is interesting to wonder whether inflammatory dysregulation could affect the qualitative and quantitative changes in MM on the ipsilateral side (referred to as the side of disc herniation). This proposal requires examination.

The aims of this study were (1) to determine the difference in the CSA of paraspinal muscles on the ipsilateral side and the unilateral side; (2) to evaluate whether the CSA of paraspinal muscles differs between individuals with disc degeneration and no degeneration; (3) to investigate whether the expression of genes for inflammatory marker(s) in MM differed between individuals with low and high fatty infiltration and was associated with the change in CSA in MM; and (4) to determine the relationship between the change in CSA/expression of genes for inflammatory marker(s) and clinical outcomes.

## Materials and methods

### Study design

Participants with chronic LBP and sciatica undergoing percutaneous endoscopic lumbar discectomy (PELD) surgery for LDH were enrolled in this prospective cohort study, which was approved by the Medical Research Ethics Committee. All experiments were performed with the approval of Institutional Medical Research Ethics Committee where participants had consented for surgically discarded tissue to be used for research. All participants consented to the use of their demographic data, radiological data, clinical scores, and paraspinal tissues for research.

### Participants

A total of 60 participants with LDH who underwent PELD surgery for sciatica with chronic LBP from January 2020 to January 2021 were included. All the participants completed at least twelve months of follow-up following PELD surgery.

The inclusion criteria were as follows: (1) aged ≥ 18 years old; (2) herniated disc in the lumbar spine region L4-L5 as confirmed by magnetic resonance imaging (MRI); (3) clinical history (e.g., sciatica with LBP) and physical examination (e.g., straight-leg-raising test) consistent with the findings on CT or MRI; and (4) nonresponsive to at least three months of nonsurgical treatment on the pain.

The exclusion criteria were as follows: (1) history of spinal deformity, tumor, infection, spondylolisthesis, and cauda equina syndrome; (2) history of lumbar spine surgery (fusion, laminectomy, or discectomy); (3) previous and current use of hormones; (4) severe organic disease, systemic metabolic bone disease, lipodystrophy, and neuromuscular syndromes; and (5) declined to participate in the project.

### Demographic data

Demographic data of the patients’ age, sex, body mass index (BMI), and duration of symptoms were collected.

### Clinical assessment

After obtaining written consent, the participants were asked to complete two questionnaires at the time of preoperative and last follow-up postoperative screening. The questionnaires are: (1) Visual Analogue Scale (VAS; 0 - no pain; 10 - worst pain imaginable) of back pain and leg pain, and (2) Oswestry disability index (ODI; a validated tool for assessing function and disability on 10 items, each item was manually rated with 5 points for six possible responses (the first statement is marked the section score = 0; the last statement is marked the section score = 5), giving a potential score between 0 and 100%.

### MRI acquisition

All participants’ MR images were obtained with a 3.0 T Trio Tim scanner (Siemens, Erlangen, Germany), and participants were positioned supine in the MRI device. Sagittal T2-weighted fast spin-echo (FSE), sagittal T1-weighted FSE, and axial T2-weighted scans were performed. The field of view (FOV), repetition time (TR)/echo time (TE), matrix size, slice thickness, slice per slab, and number of excitations (NEX) were 310 * 310 mm, 550 ms/9.6 ms, 320 * 320, 4.0 mm, 11, and 2, respectively, during the sagittal T1-weighted scan. The FOV, TR/TE, matrix size, slice thickness, slice per slab, and NEX were 310 * 310 mm, 2700 ms/97 ms, 320 * 320, 4.0 mm, 11, and 2, respectively, during the sagittal T2-weighted scan. The FOV, TR/TE, matrix size, slice thickness, slice per slab, and NEX were 210 * 210 mm, 3400 ms/102 ms, 320 * 320, 4.0 mm, 15, and 2, respectively, during the axial T2-weighted scan.

### Imaging assessment

The Pfirrmann score was used to evaluate IVD degeneration [7]. Pfirrmann grade ≥ 3 was defined as disc degeneration [13], which was used to allocate the participants into the IVD degeneration (+) and non-degeneration (-).

The Kjaer method on the MRI scans was used to evaluate fat infiltration in MM [1]. Scores were allocated as “normal/mild” for estimates of 0–10% fat and fibrous tissue within the muscle, “slight” for 10–50% fat, and “severe” for > 50% fat. All the participants were allocated into low (normal/mild + slight) and high (severe) fat infiltration groups.

**Fig. 1:**
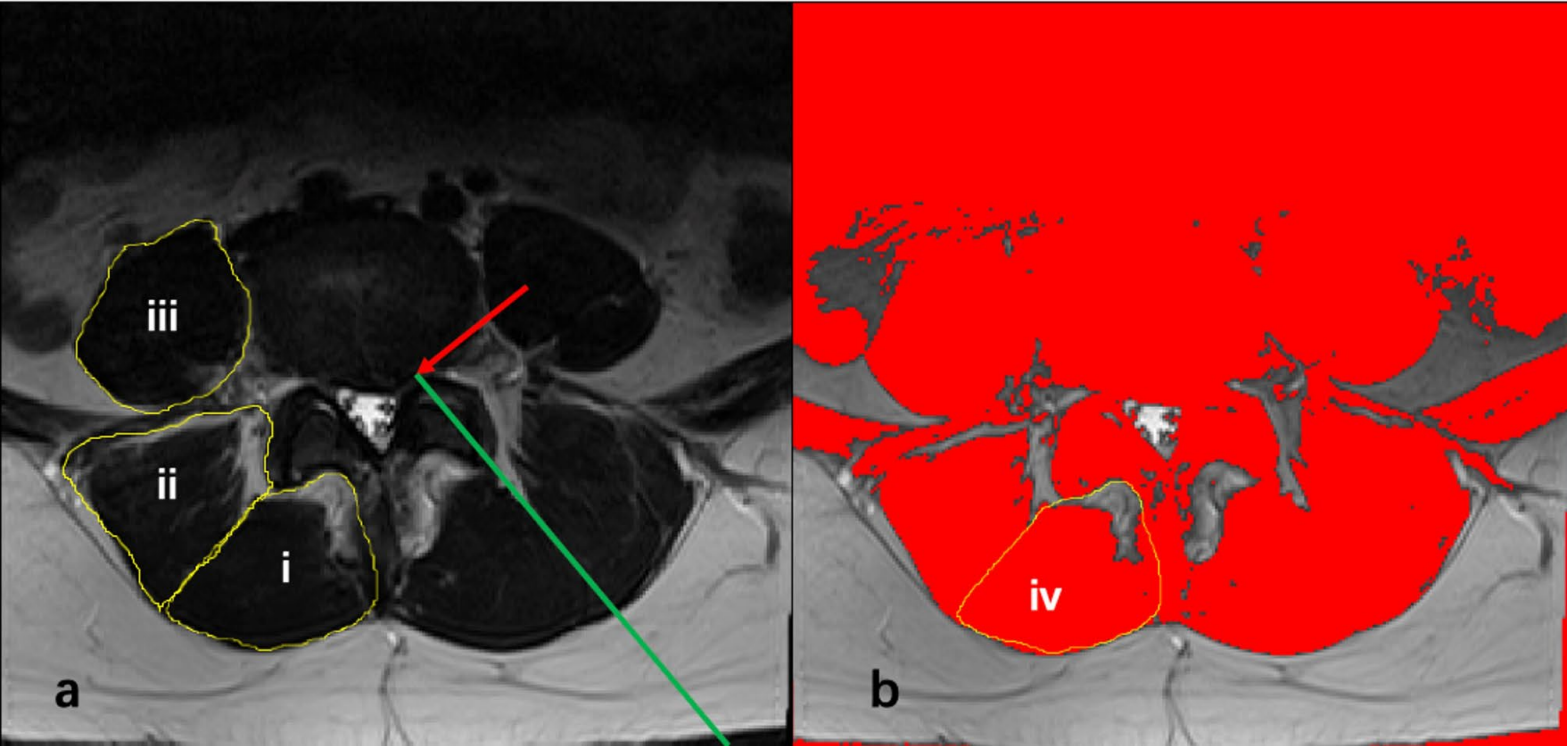
A 48-year-old male with disc herniation at L4-L5 (red arrow) (a) The CSA of the paraspinal muscles was measured by drawing the outline of the muscle fascial boundary using the range of interest (ROI) at the L4-L5 intervertebral disc level on axial T2-weighted MRI. (i) ROI of multifidus muscle; (ii) ROI of erector spinae muscle; (iii) ROI of psoas. (b) Measurement of the fatty degeneration of the multifidus muscle was performed using a threshold method. The procedure of percutaneous endoscopic lumbar discectomy (PELD) for treating disc herniation at the left side of the L4-L5 intervertebral disc level on axial T2-weighted MRI (blue line)

ImageJ software (version 1.53, National Institutes of Health, Bethesda, MD) was used to measure the quantitative measurements of paraspinal muscles, including the total (referring to the total muscles and fat) CSAs, functional (referring to fat-free) CSAs, and fatty degeneration of paraspinal muscles on the ipsilateral side (referring to the side of leg pain due to the compression of the nerve root by the herniation disc) and unilateral side. The total CSAs of the psoas muscle, MM, and erector spinae muscle were drawn from the outline of the muscle fascial boundary using the region of interest (ROI) at the L4-5 IVD level with axial T2-weighted MRI (Fig. 1). Functional CSA was calculated within the total CSA according to the method described by Fortin and Jeon et al. [14]. The ratio of functional CSA to total CSA was used to evaluate the fatty degeneration of the paraspinal muscles.

### Sample collection

A standard surgical approach was used for PELD through an endoscope (SPINENDOS GmbH, Munich, Germany). After exposing the lumbodorsal fascia, the muscle samples were harvested from the transversospinal corner of the deep MM from the ipsilateral side at the lumbar spine level of L4-L5 or L5-S1 via the endoscope according to Fig. 1. The samples were washed three times with PBS and placed in RNA later for storage at -20 °C. Once completing the sample collection, the endoscope was inserted into the herniation disc through the foraminal at the L4-L5 or L5-S1 IVD level.

### Quantitative polymerase chain reaction (qPCR) assay and inflammatory markers

RNA was extracted from muscle using RNeasy Lipid Tissue Mini Kit and RNeasy Fibrous Tissue Mini Kit (QIAGEN, Germany), and cDNA was synthesized and purified using QuantiTech Reverse transcription kit (QIAGEN, Germany). The expression of inflammatory markers in MM was examined by qPCR and normalized to glyceraldehyde 3-phosphate dehydrogenase (GAPDH) transcript, which includes TNF, interleukin-1 beta (IL-1β), IL-15, and transforming growth factor beta 1 (TGF-β1) [12, 15] (Table 1).

**Table 1:** Cytokines and molecules involved in muscle and primer sequences used for quantitative polymerase chain reaction (qPCR) analysis

Gene	Full name	Primer Sequences	Role	Function in Muscle
**TNF**	Tumor Necrosis Factor	F: 5’-GAGGCCAAGCCCTGGTATG-3’ R: 5’-CGGGCCGATTGATCTCAGC-3’	Pro-inflammatory cytokine (polypeptide cytokine)	TNF disrupts the differentiation process and can promote catabolism in mature cells which is mediated by reactive oxygen species and nuclear factor-κB for muscle wasting and weakness in inflammatory disease. TNF promotes muscle adaptation and fast muscle fiber expression.
**IL-1β**	Interleukin 1 Beta	F: 5’-AGCTACGAATCTCCGACCAC-3’ R: 5’-CGTTATCCCATGTGTCGAAGAA-3’	Pro-inflammatory cytokine	IL-1β is inhibitory towards muscle differentiation. Role in early phases of myogenesis and reduction of fibrosis
**IL-15**	Interleukin 15	F: 5’-GCCATAGCCAGCTCTTCTTCA-3’ R: 5’-CTGCACTGAAACAGCCCAAA-3’	Pleiotropic cytokine	As an anabolic factor in muscle growth, IL-15 induces an accumulation of myosin heavy chain (MHC) protein in differentiated myotubes. Play a role in muscle–adipose tissue interaction
**TGF-β1**	Transforming Growth Factor Beta 1	F: 5’-GGCCAGATCCTGTCCAAGC-3’ R: 5’-GTGGGTTTCCACCATTAGCAC-3’	Anti-inflammatory mediator	TGF-β isoforms are cytokines involved in a variety of cellular processes, including myofiber repair and regulation of connective tissue formation. Promotes atrophy/slow-to-fast transformation and induces differentiation of myocytes into myofibroblasts

F - forward primer, R - reverse primer

### Statistical analysis

All data are presented as the mean ± standard deviation (SD). Independent t-tests were used to compare quantitative measurements of paraspinal muscles and the expression of genetic markers between the disc degeneration and non-degeneration groups and between the low- and high-fat infiltration groups. A paired t-test was used to compare the clinical outcomes of VAS LBP and leg pain and ODI between preoperative and 12 months post-operative follow-up and the CSAs of paraspinal muscles, functional CSA and fatty degeneration of MM between ipsilateral and unilateral sides.

The normality of variables was evaluated. Pearson’s correlation analysis was used to test the relationships between the demographic data (including age, BMI and duration of pain), preoperative clinical scores (VAS LBP, VAS leg pain, and ODI), and expression of inflammatory markers in MM at the time of PELD surgery and the quantitative measurement data (including the total CSAs of psoas muscle, MM, and erector spinae muscle, and functional CSA and fatty degeneration of MM) on the ipsilateral side. Correlations less than 0.3, between 0.3 and 0.5, between 0.5 and 0.7, and greater than 0.7 are indicative of weak, moderate, strong, and very strong.

Interrater reliability was evaluated with intraclass correlation coefficients (ICCs) and their 95% confidence intervals (95% CIs). Values of ICC less than 0.5, between 0.5 and 0.75, between 0.75 and 0.9, and greater than 0.90 are indicative of poor, moderate, good, and excellent reliability, respectively [16]. SPSS v24.0 (SPSS Inc., Chicago, IL., USA) was used for the statistical analysis. A P value less than 0.05 was considered statistically significant.

## Results

### Patient characteristics

A total of 60 patients with LDH (37 males and 23 females; age of 43.05 ± 10.88 years, range from 20 to 65 years; BMI 27.67 ± 3.56 kg/m^2^) who underwent PELD surgery were enrolled. The mean duration of pain was 33.41 weeks (range from 70 to 14 weeks). All patients were diagnosed with single-level herniation at L4-L5. Disc degeneration and no degeneration were diagnosed in 36 and 24 patients, respectively, by using the Pfirrmann grade. Low fat infiltration was diagnosed in 29 patients. As shown in Table 2, leg pain on the right side was detected in 24 patients, and leg pain on the left side was explored in 36 patients. The mean VAS LBP, VAS leg pain, and ODI scores were 7.82 ± 1.81, 7.42 ± 1.89, and 26.64 ± 9.90 preoperatively, respectively. All the participants completed 12 months follow-up.

**Table 2:** Demographic data of included patients

Characteristic	Value
**Number of patients**	60
**Sex (male : female)**	37 (61.7%) : 23 (38.3%)
**Age (years)**	43.05 ± 10.88
**BMI (kg/m** ^**2**^ **)**	27.67 ± 3.56
**VAS LBP**	7.82 ± 1.81
**VAS leg pain**	7.42 ± 1.89
**ODI**	26.64 ± 9.90
**Duration of pain (weeks)**	33.41 ± 7.33
**Side of leg pain**	
**Left side**	36 (60.0%)
**Right side**	24 (40.0%)
**Pfirrmann grade of disc degeneration**	
**Non-degeneration**	24 (40.0%)
**Degeneration (≥ 3)**	36 (60.0%)
**Fatty infiltration**	
**Low**	29 (48.3%)
**High**	31 (51.7%)

BMI - body mass index, VAS - visual analogue scale, LBP - low back pain, ODI – Oswestry disability index; Continuous and dichotomous data are presented as mean ± standard deviation and number (rate)

### Quantitative assessment of paraspinal muscles

The total CSAs of the psoas muscle, MM, and erector spinae muscle and the functional CSA and fatty degeneration of MM on the ipsilateral side (versus unilateral side) were 1480.69 ± 316.02 mm^2^ (versus 1528.65 ± 323.66 mm^2^), 928.35 ± 183.75 mm^2^ (versus 976.15 ± 223.98 mm^2^), 1341.90 ± 327.31 mm^2^ (versus 1445.13 ± 323.75 mm^2^), 742.87 ± 166.01 mm^2^ (versus 800.29 ± 207.51 mm^2^), and 0.80 ± 0.08 (versus 0.82 ± 0.09), respectively. The statistically significant differences in the total CSAs of the psoas muscle (P = 0.000), MM (P = 0.003), and erector spinae muscle (P = 0.000) and functional CSA (P = 0.000) and fatty degeneration of MM (P = 0.001) between the ipsilateral side and unilateral side are indicated in Table 3.

**Table 3:** Quantitative measurements of paraspinal muscles

	Ipsilateral side	Unilateral side	*P*-value	Non-degeneration	Disc degeneration	*P*-value	Low-fat infiltration	High-fat infiltration	*P*-value
**Sex**	-			8 F : 16 M	15 F : 21 M	0.594	9 F : 20 M	14 F : 17 M	0.298
**Age**				45.00 ± 10.73	41.75 ± 10.93	0.260	42.01 ± 10.62	44.86 ± 10.71	0.461
**BMI**				27.72 ± 4.21	27.63 ± 3.12	0.924	27.41 ± 3.21	27.90 ± 3.90	0.599
**CSA of psoas muscle (mm** ^**2**^ **)**	1480.69 ± 316.02	1528.65 ± 323.66	0.003^**^	2964.54 ± 430.35	3039.19 ± 736.42	0.656	3134.34 ± 697.60	2892.38 ± 542.15	0.138
**CSA of MM (mm** ^**2**^ **)**	928.35 ± 183.75	976.15 ± 223.98	0.000^***^	1864.11 ± 243.84	1931.41 ± 480.75	0.530	2124.85 ± 474.57	1869.90 ± 310.44	0.006**
**CSA of erector spinae muscle (mm** ^**2**^ **)**	1341.90 ± 327.31	1445.13 ± 323.75	0.000^***^	2828.27 ± 598.38	2759.54 ± 676.97	0.688	2777.32 ± 600.50	2796.12 ± 689.00	0.911
**Functional CSA of MM (mm** ^**2**^ **)**	742.87 ± 166.01	800.29 ± 207.51	0.000^***^	1503.89 ± 149.84	1569.34 ± 460.92	0.505	1699.47 ± 331.01	1489.19 ± 402.99	0.007**
**Fatty degeneration of MM**	0.80 ± 0.08	0.82 ± 0.09	0.001^**^	0.84 ± 0.08	0.78 ± 0.08	0.005**	0.86 ± 0.07	0.77 ± 0.08	0.001**
**VAS LBP**	-			7.92 ± 1.44	7.75 ± 2.03	0.730	7.42 ± 1.75	8.45 ± 1.88	0.000^***^
**VAS leg pain**				7.79 ± 1.79	7.17 ± 1.93	0.212	7.79 ± 1.79	8.39 ± 1.09	0.000^***^
**ODI**				24.22 ± 7.55	28.24 ± 11.01	0.125	22.09 ± 7.41	30.88 ± 10.15	0.000^***^
**TNF**				0.000098 ± 0.00008	0.000104 ± 0.000008	0.791	0.000135 ± 0.00008	0.000096 ± 0.000008	0.004**
**IL-1β**				0.000305 ± 0.00026	0.000336 ± 0.00026	0.678	0.000289 ± 0.00025	0.000357 ± 0.00027	0.371
**IL-15**				0.000523 ± 0.00060	0.000605 ± 0.00057	0.712	0.000504 ± 0.00060	0.000651 ± 0.00056	0.519
**TGF-β1**				0.11 ± 0.08	0.12 ± 0.08	0.665	0.11 ± 0.08	0.13 ± 0.08	0.486

CSA – cross-sectional area; MM – multifidus muscle; F – female; M – male; TNF – tumor necrosis factor; IL – interleukin; TGF – transforming growth factor; The ration of functional CSA of multifidus muscle to total CSA of multifidus muscle was used to evaluate the fatty degeneration of the multifidus muscle. Data are presented as mean ± standard deviation. Significant difference ** *P* < 0.01, *** *P* < 0.001 (*t*-tests)

### Comparison of quantitative data of paraspinal muscles and clinical outcomes between different disc degeneration/fat infiltration groups

Compared with the no degeneration group, participants in the disc degeneration group displayed higher fat infiltration in MM (P = 0.005). Participants with higher preoperative levels of fat infiltration in the MM displayed worse LBP (P = 0.000) and leg pain (P = 0.000) measured with the VAS, function as measured by the ODI (P = 0.000), a higher reduction of CSA of MM (P = 0.006), functional CSA (P = 0.007) and fatty degeneration of MM (P = 0.001) in the high-fat infiltration group (Table 3).

### Association between demographic data and quantitative data of paraspinal muscles

There was no correlation between demographic data (including age, BMI, and duration of pain) and quantitative data of paraspinal muscles (including the total CSAs of the psoas muscle, MM, and erector spinae muscle and functional CSA and fatty degeneration of MM) on the unilateral side.

Age showed a moderate association with fatty degeneration of MM on the ipsilateral side (r=-0.347, P = 0.007). The duration of pain had a moderate negative correlation with the CSA of MM (r=-0.368, P = 0.041) and the functional CAS of MM (r=-0.478, P = 0.022) (Table 4).

**Table 4:** Pearson’s correlation coefficient between continuous variables

	Age	BMI	Duration of pain	VAS LBP	VAS leg pain	ODI	TNF	IL-1β	IL-15	TGF-β1
**Unilateral side**										
**CSA of psoas muscle (mm** ^**2**^ **)**	0.169 (0.196)	-0.259 (0.056)	-0.081 (0.512)	-0.102 (0.438)	0.014 (0.918)	-0.252 (0.052)	-			
**CSA of multifidus muscle (mm** ^**2**^ **)**	-0.192 (0.054)	-0.017 (0.899)	-0.041 (0.714)	-0.005 (0.967)	-0.089 (0.497)	-0.144 (0.273)				
**CSA of erector spinae muscle (mm** ^**2**^ **)**	0.176 (0.178)	0.079 (0.612)	0.078 (0.552)	0.043 (0.746)	0.204 (0.118)	0.023 (0.859)				
**Functional CSA of multifidus muscle (mm** ^**2**^ **)**	0.054 (0.680)	-0.131 (0.318)	-0.121 (0.287)	-0.015 (0.907)	-0.079 (0.546)	-0.142 (0.281)				
**Fatty degeneration of multifidus muscle**	-0.175 (0.229)	0.130 (0.185)	-0.011 (0.178)	-0.004 (0.978)	0.091 (0.488)	-0.027 (0.836)				
**Ipsilateral side**										
**CSA of psoas muscle (mm** ^**2**^ **)**	0.098 (0.457)	-0.210 (0.108)	0.224 (0.176)	0.037 (0.782)	-0.112 (0.393)	-0.201 (0.084)	-0.364 (0.011*)	-0.159 (0.271)	0.183 (0.342)	0.254 (0.109)
**CSA of multifidus muscle (mm** ^**2**^ **)**	-0.281 (0.030*)	0.061 (0.643)	-0.368 (0.041*)	0.101 (0.440)	-0.228 (0.021*)	-0.244 (0.026*)	-0.509 (0.000***)	-0.674 (0.000***)	-0.058 (0.771)	-0.255 (0.107)
**CSA of erector spinae muscle (mm** ^**2**^ **)**	-0.262 (0.041*)	0.053 (0.690)	0.236 (0.173)	0.044 (0.738)	-0.260 (0.044*)	0.054 (0.680)	-0.403 (0.004**)	-0.129 (0.103)	0.252 (0.092)	0.162 (0.222)
**Functional CSA of multifidus muscle (mm** ^**2**^ **)**	-0.101 (0.441)	-0.018 (0.890)	-0.478 (0.022*)	0.091 (0.488)	-0.148 (0.260)	-0.085 (0.520)	-0.437 (0.002**)	-0.691 (0.000***)	0.062 (0.756)	0.313 (0.046*)
**Fatty degeneration of multifidus muscle**	-0.347 (0.007**)	-0.160 (0.471)	0.266 (0.321)	-0.033 (0.800)	-0.082 (0.533)	0.100 (0.446)	-0.488 (0.000**)	0.064 (0.657)	0.330 (0.087)	0.200 (0.209)
**VAS LBP**	-						0.410 (0.004**)	0.522 (0.000***)	-0.119 (0.545)	0.682 (0.000***)
**VAS leg pain**							0.286 (0.049*)	0.462 (0.001**)	0.298 (0.123)	0.387 (0.012*)
**ODI**							0.502 (0.000***)	0.449 (0.001**)	-0.028 (0.889)	0.354 (0.023*)

BMI - body mass index; VAS - visual analogue scale, LBP - low back pain, ODI – Oswestry disability index, CSA – cross-sectional area; TNF – tumor necrosis factor; IL – interleukin; TGF – transforming growth factor; The ration of functional CSA of multifidus muscle to total CSA of multifidus muscle was used to evaluate the fatty degeneration of the multifidus muscle. Data was presented as coefficient value (P value)

Significant difference * P < 0.05, **P < 0.01, *** P < 0.001 (Pearson’s correlation coefficient)

### Association between quantitative data of paraspinal muscles and clinical outcomes

Quantitative measurement data of paraspinal muscles (including the total CSAs of the psoas muscle, MM, and erector spinae muscle, and functional CSA and fatty degeneration of MM) on the unilateral side were not related to preoperative clinical outcomes (including VAS LBP, VAS leg pain, and ODI).

There was a negative association between the CSAs of MM (r=-0.228, P = 0.021) and the erector spinae muscle (r=-0.260, P = 0.044) on the ipsilateral side and VAS leg pain. There was a statistically significant correlation between CSA of MM on the ipsilateral side and ODI (r=-0.244, P = 0.026) (Table 4).

### Association between inflammatory markers and quantitative measurements of MM on the ipsilateral side

The expression of TNF in MM was moderately correlated with the total CSAs of the psoas muscle (r=-0.364, P = 0.011), MM (r=-0.509, P = 0.000), and erector spinae muscle (r=-0.403, P = 0.004), functional CSA (r=-0.437, P = 0.002) and fatty degeneration of MM (r=-0.488, P = 0.000). The expression of IL-1β in MM was strongly correlated with the CSA of MM (r=-0.674, P = 0.000) and the functional CSA of MM (r=-0.691, P = 0.000). The expression of TGF-β1 was moderately correlated with the functional CSA of MM (r=-0.313, P = 0.046) (Table 4).

### Comparison of inflammatory markers in the MM between different Disc Degeneration/Fat infiltration groups

TNF expression was greater in participants with high-fat infiltration than in those with low-fat infiltration (Table 3). There were no differences in the expression of IL-1β, IL-15, and TGF-β1 between the disc degeneration and no degeneration groups or between the low- and high-fat infiltration groups.

### Association between inflammatory markers and clinical outcomes

The expression of TNF, IL-1β, and TGF-β1 in MM was moderately to strongly correlated with the clinical outcomes (TNF with clinical outcome: VAS LBP (r = 0.410, P = 0.004), VAS leg pain (r = 0.286, P = 0.049), and ODI (r = 0.502, P = 0.000); IL-1β with clinical outcomes: VAS LBP (r = 0.522, P = 0.000), VAS leg pain (r = 0.462, P = 0.001), and ODI (r = 0.449, P = 0.001); TGF-β1 with clinical outcomes: VAS LBP (r = 0.682, P = 0.000), VAS leg pain (r = 0.387, P = 0.012), and ODI (r = 0.354, P = 0.023)). There was no significant correlation between the expression of IL-15 in MM and clinical outcomes (including VAS LBP, VAS leg pain, and ODI) (Table 4).

### Interrater reliability

There was good to excellent agreement in terms of interrater reliability for the quantitative measurements on the ipsilateral side (including CSA of psoas: 0.087 (0.856, 0.902), CSA of MM: 0.854 (0.839, 0.887), CSA of erector spinae muscle: 0.855 (0.841, 0.889), functional CSA of MM: 0.788 (0.771, 0.825), and fatty degeneration of MM: 0.813 (0.807, 0.854)) and unilateral side (including CSA of psoas: 0.901 (0.889, 0.928), CSA of MM 0.894 (0.876, 0.923), CSA of erector spinae muscle: 0.894 (0.876, 0.915), functional CSA of MM: 0.832 (0.804, 0.867), and fatty degeneration of MM: 0.845 (0.823, 0.888)).

## Discussion

The results of this study provide evidence of the quantitative changes in paraspinal muscles between the ipsilateral side and unilateral side and a relationship between the CSA of paraspinal muscles and CSA of lean muscle and fatty in MM and inflammatory dysregulation in the local environment in patients with LDH. Data show a significant reduction in the CSAs of the paraspinal muscles on the ipsilateral sciatica only. There is evidence for the relationships between MM dysfunction and poor functional outcome preoperatively and between fat accumulation in MM and disc degeneration/inflammatory dysregulation in the local environment. Data also show a greater proinflammatory response in MM in individuals with high-fat infiltration. TNF is moderately associated with the quantitative measurements (the total CSAs of the psoas muscle, MM, and erector spinae muscle, and functional CSA and fatty degeneration of MM) on the ipsilateral side. Upregulation of a greater proinflammatory response in the MM in individuals with low CSAs and high-fat infiltration. These findings have potential implications for understanding the mechanisms underlying the paraspinal muscle changes associated with disc herniation in humans relative to animal models.

### Quantitative data of paraspinal muscles on the ipsilateral and unilateral sides

LDH is associated with paraspinal muscle morphological changes comprising the size, type, and distribution of fibers, especially changes in MM [17]. The medial branch of the dorsal ramus of the segmental nerve innervated the paraspinal muscle, which could lead to structural changes in paraspinal muscles due to denervation, disuse, or an inflammatory response [3, 11, 18–20]. The lesion of the compressive nerve root by the herniated disc led to muscle fiber denervation, which could cause quantitative changes in the paraspinal muscles. Additionally, the persistent compression of the nerve root contributes to fatty infiltration and atrophy of muscle fibers supplied by that nerve [17]. Furthermore, increased fatty infiltration of MM occurred with a consistent reduction in muscle CSA. Taken together, these cascade actions indicate that individuals with LDH might already undergo a structural change in the paraspinal muscles [18, 21]. Of note, there was a significant reduction in the CSAs of paraspinal muscles on the ipsilateral side compared to the unilateral side [21–23], which is consistent with our results. It is interesting to wonder what the main mechanism for causing the structural changes of paraspinal muscles is and whether these changes will affect the clinical outcomes.

Spontaneous IVD degeneration or IVD injury can cause inflammatory dysregulation in the local environment and mechanical changes in the lumbar spine, which is considered to be the leading cause of the structural changes in paraspinal muscles [2, 4–6, 11, 24]. Although the LBP rat model has provided evidence for the relationship between IVD degeneration and fat infiltration of paraspinal muscles [25], the direct causal relationship between IVD degeneration and paraspinal muscle fatty infiltration and quantitative measurements is unclear. Our study showed that there was no significant association between disc degeneration and quantitative measurements on paraspinal muscle. One of the main explanations is that most of the patients who underwent PELD surgery for LDH had disc degeneration (Pfirrmann grade ≥ 3), which potentially affected the results.

### Association between the changes in paraspinal muscles and clinical outcomes

At present, multiple studies have reported an association between the morphological changes of paraspinal muscles and the presence of LBP and leg pain and functional limitation, which are influenced by the duration of pain [26–30]. However, due to a lack of measurement at a specific level and ipsilaterally to the pain source, the notion that MM morphological changes are not strong predictors of LBP or leg pain is also supported by some earlier studies, which failed to detect the associations between structural changes in paraspinal muscles and the presence of LBP or the duration of existing LBP symptoms [31, 32]. The results of the present study provide evidence for the relationship between paraspinal muscle dysfunction and poor clinical outcomes (e.g., LBP, leg pain, and disability).

### Proinflammatory cytokine expression in MM

These data showed that the greater expression of the pro-inflammatory cytokine TNF was elevated in MM of LDH patients with a high fatty infiltration (a high fatty degeneration rate) and reduction in the CSA of muscles, which provided evidence for explaining the bidirectional relationship between fat and TNF. Hence, it was supported that adipose tissue is the main source or promoter of TNF expression, and as the driver of adipogenesis, TNF expression also accelerated fat accumulation. Animal models of disc degeneration/injury have identified that the increased proportion of proinflammatory M1 macrophages due to greater TNF expression is significantly associated with fat and connective tissue accumulation in MM [2, 33]. The absence of TNF in human MM underlies the increased fibrotic activity in MM during chronic LBP [12]. A previous human study provided evidence for greater TNF expression in MM of LDH patients with high fatty infiltration [12]. Taken together, TNF from alternate sources promotes fat infiltration in the human context, rather than the converse.

IL-1β is a potent proinflammatory cytokine critical to multiple pathologies in the paraspinal muscles. Greater expression of IL-1β promotes muscle differentiation and plays a role in the early phases of myogenesis and reduction of fibrosis, which could lead to the reduction of CSA of muscle [10]. An animal study on an IVD lesion model showed that blocking the expression of IL-1β by exercise could have diverse fibrosis/fatty infiltration, and reduced IL-1β in MM after mesenchymal stem cell treatment is associated with attenuated/delayed development of the components of structural remodelling present in MM [5]. TGF-β isoforms are cytokines involved in a variety of cellular processes, including myofiber repair and regulation of connective tissue formation. TGF-β plays an essential role in the development of tissue fibrosis and is a molecular marker in the study of muscle fibrosis, which has been reported in the MM of LDH patients [34]. Greater expression of TGF-β promotes atrophy/slow-to-fast transformation and induces differentiation of myocytes into myofibroblasts. IL-15 is a skeletal muscle-derived cytokine with favourable effects on muscle mass and body composition. As an anabolic factor in muscle growth, greater expression of IL-15 induces an accumulation of myosin heavy chain (MHC) protein in differentiated myotubes and plays a role in muscle–adipose tissue interactions [35]. The changes in cytokine expression in MM in our study supported the translation of the findings from animal models to humans.

### Proinflammatory cytokine expression with clinical outcome

Systemically, the expression of proinflammatory cytokines is increasingly recognized in chronic pain conditions, such as the elevation of TNF and IL-1β in chronic LBP. Previous studies supported that proinflammatory cytokines (including TNF and IL-1β) from adipose tissue have been implicated in the association between obesity and osteoarthritis [36, 37]. In obesity, paraspinal adipose tissue is considered the source of systemic proinflammatory cytokines, which could drive the accumulation and/or polarization of adipose tissue macrophages to low-grade chronic inflammation. Our investigation of the inflammatory state of MM revealed that local TNF and IL-1β expression could help explain both chronic pians (LBP and radicular pain) and provide an alternative mechanism for the quantitative changes of muscles in some individuals. Similar to our study, a recent study showed that the elevation of TNF and IL-1β in paraspinal tissues (including muscles and fat) in LDH patients was associated with high-fat infiltration with poor postoperative outcome [12, 15]. Moreover, a study showed that the upgraded expression of TNF in serum was associated with poor recovery in patients with LBP plus sciatica or not [38]. A previous study provided evidence supporting IL-1β as a potent inflammatory cytokine involved in the mechanism of allodynia and possibly in the development of postoperative chronic pain [15], which is consistent with our results. TGF-β1 was also reported to be a cytokine that plays an essential role in the development of tissue fibrosis and is a molecular marker in the study of muscle fibrosis. A clinical study showed that nerve root compression by LDH leads to multifidus atrophy, fibrosis, and increased TGF-β1 expression, which promote MM fibrosis and are associated with changes in pain and disability scores [34]. Although our study provided evidence for the cascade actions of the reduction of CSA in paraspinal muscles in patients with LDH and the association of high expression of proinflammatory cytokines and the reduction of CSA of paraspinal muscles with poor clinical outcomes, the potential underlying mechanisms remain unclear and warrant further investigation.

### Methodological issues

There are also some shortcomings identified in this study that require a brief discussion. First, healthy participants as a control group are missing. Second, potential bias during sample collection during PELD surgery exists. The tissue samples collected for this study were only harvested from the ipsilateral side during PELD surgery. Despite all care in selecting areas to collect samples from, there is a possibility of tissue sampling error due to the difference between ipsilateral and unilateral sides. Third, only one T2-axial image on the level of L4-L5 was used for measuring the CSA of paraspinal muscles. Finally, the expression of the proteins was not measured. Future prospective studies should investigate the expression of marker(s) in paraspinal muscles using a randomized controlled design with a larger sample size.

## Conclusion

This study demonstrated that there were differences in the characteristics of paraspinal muscles between the ipsilateral and unilateral sides in patients with LDH, which was affected by disc degeneration and the degree of fat infiltration in MM. Furthermore, the novel results presented here support the hypothesis that the high-fat filtration and reduction of CSA of MM are associated with inflammatory dysfunction. Finally, there was evidence of a dysregulated inflammatory profile in MM in individuals with poor clinical outcomes.

## Data Availability

The datasets used and/or analysed during the current study available from the corresponding author (Xiaolong Chen) on reasonable request.
